# Harley Street

**Published:** 1921-06-04

**Authors:** 


### HARLEY STREET.

Since the close of the war period, among the many trials of the younger men attached to the medical staffs of the London hospitals have been not only the difficulty of obtaining a W. 1 consulting-room for the practice they are setting out to build, but also the exorbitant rentals demanded in the approved medical streets. Apart from the general boom in prices, the situation arose with an influx of provincial and ex-Army doctors who, somewhat speculatively, paid high rents for quarters by long custom reserved for those with hospital appointments. Nowadays the slump in all professional work is driving home the truth that an address in Harley Street is not of itself an "open sesame" to fame and fortune. Consequently there is a steady flow of departures to less expensive neighbourhoods and, for these newcomers, the more lucrative paths of general practice. Soon the young consultant should find the position entirely changed.

The downward tendency in rentals is already noticeable, but, without waiting for this automatic levelling to occur, would it not be possible for one or other of the bodies representative of the consultant medical profession to regularise the position, or at least exert some degree of censorship over the prices asked? Admittedly the owner of the house is to some extent entitled to ask and obtain the best figure he can, but this should not, as now frequently obtains, be grossly in excess of all the facilities provided.

There are, of course, exceptions. One knows many of them, but still there are the notoriously high prices of the last two years to be remembered, and our criticism must fall where it is deserved.

